# Enhanced Quality and Metabolic Profile of Fermented Milk Through Fucose Supplementation with *Lactobacillus helveticus*

**DOI:** 10.3390/molecules31060990

**Published:** 2026-03-16

**Authors:** Shunyu Wang, Hongchao Wang, Yurong Zhao, Zhangming Pei, Wenwei Lu, Jianxin Zhao, Shourong Lu

**Affiliations:** 1 State Key Laboratory of Food Science and Resources, Jiangnan University, Wuxi 214122, China; 7200112089@stu.jiangnan.edu.cn (S.W.); 6230111151@stu.jiangnan.edu.cn (Y.Z.); peizhangming@jiangnan.edu.cn (Z.P.); luwenwei@jiangnan.edu.cn (W.L.); zhaojianxin@jiangnan.edu.cn (J.Z.); 2 School of Food Science and Technology, Jiangnan University, Wuxi 214122, China; 3 Zhejiang Liziyuan Food Co., Ltd., Jinhua 321031, China; 4 The Affiliated Wuxi People’s Hospital of Nanjing Medical University, Wuxi People’s Hospital, Wuxi Medical Center, Nanjing Medical University, Wuxi 214122, China; lushourong@njmu.edu.cn

**Keywords:** microbial fermentation, fucose, metabolomics, probiotics

## Abstract

Fermented milk represents an excellent carrier for probiotics, and the incorporation of different carbon sources during fermentation can profoundly affect microbial metabolism. Based on our previous finding that *Lactobacillus helveticus* DYNDL_20-5 produces fucose-containing exopolysaccharides (EPS), we hypothesized that fucose supplementation could further enhance its metabolic activity and improve fermented milk quality. Thus, this study systematically investigated the impact of culturing *L*. *helveticus* DYNDL_20-5 with fucose (CMF5) on the quality characteristics and metabolic profiles of fermented milk. Compared to the control group without fucose and *L. helveticus* (CM), the CMF5 group demonstrated that *L. helveticus* effectively utilized fucose to promote acid production, enhance the fermentation process, increase microbial abundance, and enrich beneficial genera. Furthermore, the CMF5 group exhibited significantly improved textural properties, including enhanced viscosity and gel strength. Metabolomic analysis revealed that the addition of fucose and *L. helveticus* significantly influenced the metabolism of organic acids, fatty acids, and amino acids during milk fermentation, leading to increased concentrations of various metabolites associated with sensory quality, nutritional value, and health-promoting benefits. The findings of this study provide valuable insights into the synergistic effects of *L. helveticus* DYNDL_20-5 and fucose on fermented milk quality, offering a theoretical foundation for the development of novel functional dairy products with enhanced nutritional and sensory attributes.

## 1. Introduction

Fermented foods represent a distinct food culture in communities worldwide, symbolizing the heritage and socio-cultural aspects of diverse populations [1]. Among these, fermented milk is produced by pasteurizing raw milk and then fermenting it with suitable and harmless microorganisms [2]. Fermented milk possesses unique functional characteristics attributed to the presence of functional microorganisms, which impart various health benefits to consumers. These microorganisms exhibit probiotic properties, as they are defined as living microorganisms that, when administered in adequate quantities, exert beneficial health effects on the host [3]. Clinical and experimental studies have established that fermented dairy products possess immunomodulatory, anti-carcinogenic, hypocholesterolemic, antioxidant, and hypotensive properties [2].

Cow milk harbors a complex microbial ecosystem, including lactic acid bacteria (LAB) such as *Lactococcus*, *Streptococcus*, and *Lactobacillus* genera [4]. Among these, *Lactobacillus helveticus* (*L. helveticus*) is frequently employed in the production of fermented milk owing to its ability to prevent bitterness through proteolytic activity, its probiotic effects, and its health-enhancing properties [5,6]. *L. helveticus* is a Gram-positive, rod-shaped lactic acid bacterium [7]. It is classified as an industrial homofermentative, thermophilic, non-spore-forming starter bacterium with generally recognized as safe status and an optimum growth temperature of 42 °C [8]. The fermentation process of *Lactobacillus*-fermented milk primarily depends on the metabolic activity of the bacteria. *L. helveticus* can ferment carbohydrates such as lactose to produce organic acids, predominantly lactic acid, thereby lowering the pH of fermented milk. This acidification causes casein to denature and coagulate, resulting in the characteristic curd formation and textural development [9]. Research has demonstrated that *L. helveticus*–fermented dairy products exert cardioprotective effects and promote digestive and immune functions [7]. Furthermore, the exopolysaccharides (EPS) produced by *L. helveticus* can serve as effective stabilizers and thickening agents, thereby enhancing the viscosity, texture, stability, and mouthfeel of fermented dairy products [10,11].

Cow milk serves as a natural habitat for LAB, and the type of carbon source can significantly influence their metabolism and EPS production [12]. Research has demonstrated that the type of carbon source affects the biomass accumulation of *Streptococcus thermophilus* [13]. Moreover, changes in the carbon source of the LAB culture medium significantly affect the yield and compositional characteristics of LAB’s EPS [14]. Torino et al. reported that *L. helveticus* ATCC 15807 cultivated with lactose as the carbon source produced higher EPS yields compared to glucose, at pH 4.5 [15]. Additionally, altering the carbon source for LAB fermentation can improve the texture, viscosity, mouthfeel, and flavour profile of fermented milk products [16].

In our previous screening of LAB, *L. helveticus* DYNDL_20-5 was identified as a strain producing fucose-containing EPS [17]. This unique feature prompted us to investigate whether supplementing fucose during fermentation could further enhance its metabolic activity and improve the quality of fermented milk. We hypothesized that fucose supplementation would modulate the metabolic profile of *L. helveticus* DYNDL_20-5 during milk fermentation, thereby improving the textural properties, nutritional value, and functional characteristics of the resulting product. L-Fucose (6-deoxy-L-galactose) is a rare monosaccharide that differs from most D-monosaccharides by the absence of a hydroxyl group at the C-6 position [18]. It presents as a white crystalline powder that is readily hygroscopic, possessing a hydrophilicity lower than that of other monosaccharides [19]. L-Fucose possesses significant physiological functions, including anti-cancer, anti-allergic, anticoagulant, and anti-ageing effects [20]. Its anti-cancer potential is mediated by the modulation of fucosylation through dietary intake. Additionally, it slows skin aging by regulating collagen and non-collagen protein biosynthesis, modifying collagen accumulation in fibroblasts [21]. Previous studies have demonstrated that *Lacticaseibacillus rhamnosus* GG can metabolize fucose to generate energy and support cellular proliferation [22].

Currently, there is limited research on the role of *L. helveticus* in metabolizing fucose to enhance the quality characteristics and functional properties of fermented milk. Therefore, this study was conducted to provide a comprehensive theoretical basis for understanding the impact of *L. helveticus* DYNDL_20-5 on the quality characteristics, metabolic profiles, and functional attributes of fermented milk during fermentation in the presence of fucose.

## 2. Results

### 2.1. Strain Characteristics

Through the multi-step screening process described in Section 4.2, *L. helveticus* DYNDL_20-5 was identified as a high EPS-producing strain. Monosaccharide composition analysis revealed that the EPS produced by this strain contained fucose, a feature that distinguished it from other high-producing strains. Therefore, *L. helveticus* DYNDL_20-5 was selected for further investigation in fermented milk. Further details on the screening results can be found in our previous work [17].

### 2.2. pH and Titratable Acidity

**Figure 1:**
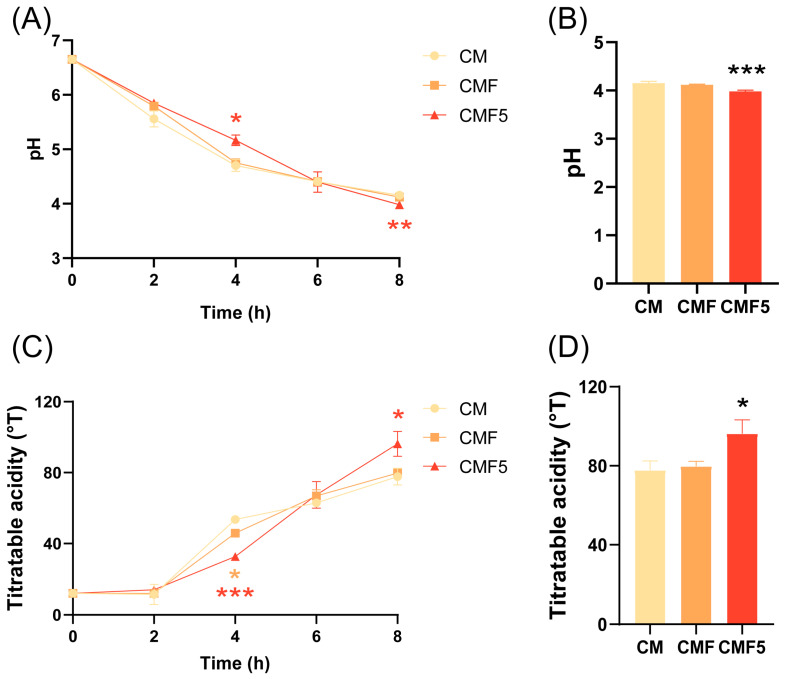
pH change (**A**), pH value at fermentation endpoint (**B**), acidity change (**C**) and acidity value at fermentation endpoint (**D**) of fermented milk. Error bars represent ± SEM. * *p* < 0.05, ** *p* < 0.01, *** *p* < 0.001. One-way ANOVA with Dunnett’s multiple comparison test in (**B**,**D**), and two-way ANOVA with Tukey’s multiple comparison test in (**A**,**C**).

Changes in pH and titratable acidity were monitored throughout the fermentation process in three experimental groups of fermented milk (Figure 1A–D). A two-way analysis of variance (ANOVA) with Tukey’s multiple comparison test was performed to assess the effects of fermentation time and experimental group on pH and titratable acidity (TA) dynamics. After 2 h of fermentation, the pH of the milk began to decrease rapidly, with the CM and CMF groups exhibiting a faster rate of decline compared with the CMF5 group. At 4 h of fermentation, the pH of CMF5 was significantly higher than that of CM (*p* < 0.05); and TA of CMF and CMF5 was significantly lower than that of CM (*p* < 0.05), indicating a delayed acidification rate in these two groups. At the fermentation endpoint, the pH values of all three groups had decreased below 4.5, with the CMF5 group demonstrating a significantly lower pH than the CM group (*p* < 0.001). Concurrently, the lactic acid content in the CMF5 group was significantly higher than in the CM group (*p* < 0.05), indicating enhanced acidification capacity.

### 2.3. Water Holding Capacity and Absolute Quantitation of Microbiota Abundance

**Figure 2:**
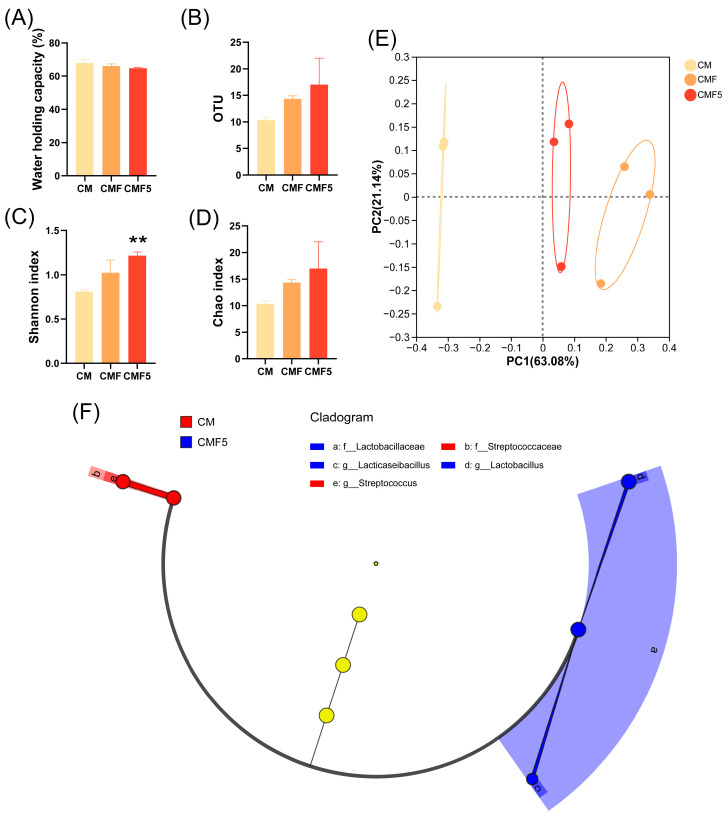
Water-holding capacity (**A**), operational taxonomic units (OTUs) (**B**), Shannon index (**C**), Chao index (**D**), principal coordinate analysis (PCoA) plot (**E**), and linear discriminant analysis Effect Size (LEfSe) analysis (**F**) at the fermentation end point of fermented milk. Yellow circles represent microbial taxa with no significant differences. Error bars represent ± SEM. ** *p* < 0.01. One-way ANOVA with Dunnett’s multiple comparison test in (**A**–**D**).

The water holding capacity (WHC) of fermented milk is presented in Figure 2A. No significant differences were observed in the WHC amongst the three groups; however, a slight decrease in WHC was noted in the CMF5 group.

A total of 10, 14, and 17 distinct bacterial operational taxonomic units (OTUs) were identified in the CM, CMF, and CMF5 groups, respectively, as illustrated in Figure 2B. To further elucidate the bacterial compositional differences amongst these three fermented dairy product groups, comprehensive α-diversity analysis was conducted (Figure 2C,D). Compared with the CM group, the Shannon index in the CMF5 group exhibited a statistically significant increase (*p* < 0.01), whilst the Chao index demonstrated no significant variation.

β-diversity analysis enables the determination of microbial community dissimilarity between different sample groups [23]. The principal coordinate analysis (PCoA) plot (Figure 2E), generated based on the fermented milk microbiome data, exhibited distinct group-based clustering patterns with clear spatial separation. Specifically, the CM group distributed predominantly within the left quadrant, whilst the CMF and CMF5 groups were primarily positioned within the right quadrants of the PCoA plot.

Linear discriminant analysis Effect Size (LEfSe) represents a robust statistical approach commonly employed to identify and analyse differentially abundant microbial taxa between experimental groups. Through LEfSe analysis comparing the CM and CMF5 groups, we successfully identified the differentially abundant bacterial genera characterising each treatment. At the species level, the CM group was predominantly characterised by *Streptococcus*, which is consistent with the composition of the commercial starter culture (containing *Streptococcus thermophilus*). In contrast, the CMF5 group exhibited enrichment of *Lactobacillaceae*. At the genus level, the differential taxon in the CM group was *Streptococcaceae*, whereas the CMF5 group demonstrated enrichment of *Lacticaseibacillus* and *Lactobacillus* genera.

### 2.4. Textural and Rheological Properties

**Figure 3:**
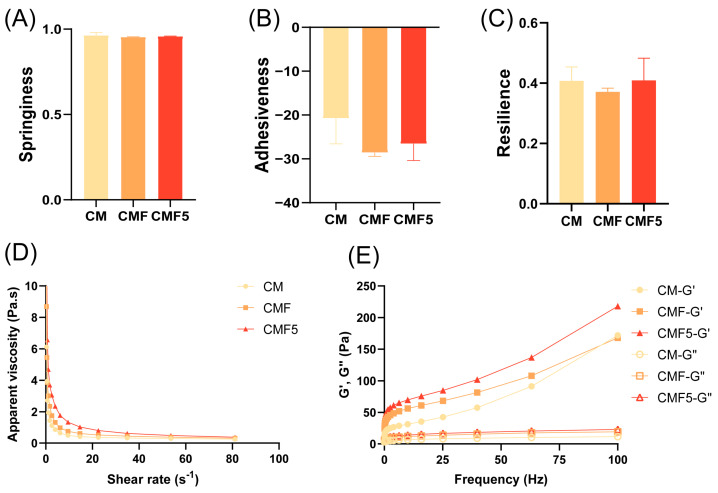
Springiness (**A**), adhesiveness (**B**), resilience (**C**), apparent viscosity (**D**), and storage modulus (G′) and loss modulus (G″) (**E**) at the fermentation end point of fermented milk. Error bars represent ± SEM. One-way ANOVA with Dunnett’s multiple comparison test in (**A**–**C**).

The textural characteristics of fermented milk products are of paramount importance, as they play a vital role in determining consumer acceptance and overall product quality. In the present study, springiness, adhesiveness, and resilience were evaluated to comprehensively characterise the texture profiles of fermented milk samples. No statistically significant differences were observed in springiness, resilience, or cohesiveness amongst the experimental groups (Figure 3A–C), suggesting that these textural parameters remained relatively stable across treatments.

The apparent viscosity of fermented milk serves as a critical indicator reflecting sample stability, with higher viscosity values corresponding to enhanced product stability. As demonstrated in Figure 3D, within the shear rate range of 1–100 s^−1^, the apparent viscosity of fermented milk decreased progressively with increasing shear rate, exhibiting characteristic shear-thinning behaviour. This rheological response indicates that fermented milk displays pseudoplastic fluid characteristics, confirming its classification as a non-Newtonian fluid [24].

Within the frequency scanning range employed, both the storage modulus (G′) and loss modulus (G″) of all samples increased in direct proportion to the scanning frequency, exhibiting characteristic frequency-dependent behaviour (Figure 3E). Notably, G′ consistently exceeded G″ across all frequency ranges examined. The G′ and G″ values of the CMF group were significantly higher than those observed in the other experimental groups.

### 2.5. Non-Targeted Metabolomic Analysis

**Figure 4:**
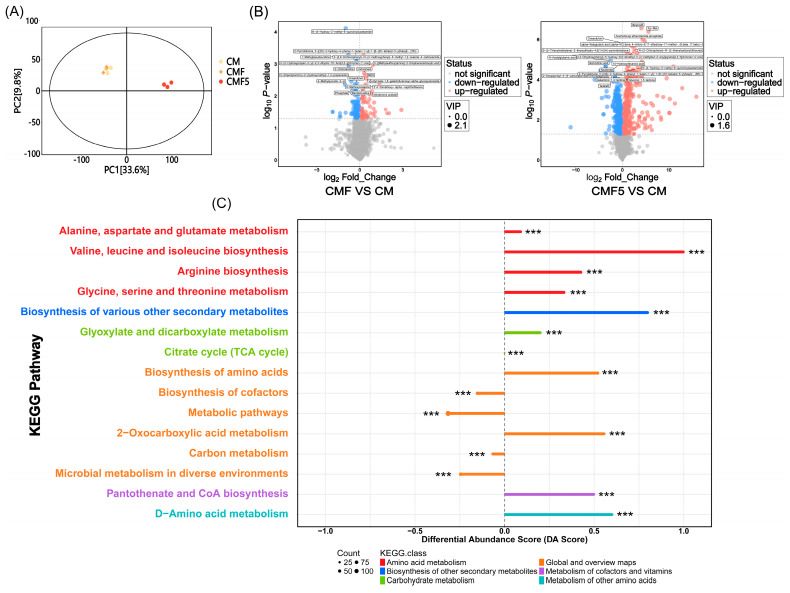
PCA plot (**A**), volcano plot (**B**) and KEGG enrichment pathway analysis (**C**) at fermentation end point of fermented milk. *** *p* < 0.001.

Changes in the metabolic profile of fermented milk supplemented with fucose and *L. helveticus* DYNDL_20-5 were comprehensively investigated using a non-targeted metabolomics approach. On the principal component analysis (PCA) score plot, a distinct separation trend was observed between the CM and CMF5 samples, with each group occupying discrete regions of the multivariate space, while no distinct separation between the CM and CMF samples (Figure 4A).

Amongst the detected metabolites, comparative analysis revealed that the CMF group exhibited 93 metabolites significantly downregulated and 60 metabolites significantly upregulated relative to the CM group. In comparison with the CM group, the CMF5 group demonstrated 519 and 311 metabolites that were significantly downregulated and upregulated, respectively, indicating a more pronounced metabolic shift. Amongst the significantly increased differential metabolites were ifenprodil, tryptophan-methionine (Trp-Met) dipeptide, and arachidonoyl ethanolamide phosphate (Figure 4B).

Further KEGG pathway enrichment analysis was conducted on CMF5 fermented milk to elucidate the metabolic alterations induced by fucose metabolism. As anticipated, *L. helveticus* DYNDL_20-5 demonstrated significant fucose-metabolising capacity, which profoundly influenced amino acid metabolism in the fermented milk matrix, particularly affecting the biosynthetic pathways of branched-chain amino acids (BCAAs), namely valine, leucine, and isoleucine (Figure 4C).

### 2.6. Alterations in Amino Acid, Organic Acid, and Fatty Acid Metabolic Profiles

**Figure 5:**
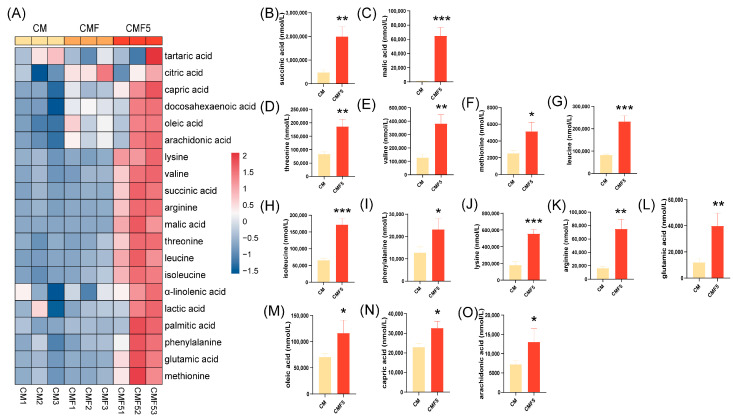
Heatmap of free amino acids (FAAs), free fatty acids (FFAs) and organic acid levels (**A**), succinic acid level (**B**), malic acid level (**C**), threonine level (**D**), valine level (**E**), methionine level (**F**), leucine level (**G**), isoleucine level (**H**), phenylalanine level (**I**), lysine level (**J**), arginine level (**K**), glutamic acid level (**L**), oleic acid level (**M**), capric acid level (**N**) and arachidonic acid level (**O**) at fermentation end point of fermented milk. Error bars represent ± SEM. * *p* < 0.05, ** *p* < 0.01, *** *p* < 0.001. Student’s *t*-test in (**B**–**O**).

Given that LAB fermentation of dairy substrates generates substantial quantities of bioactive metabolites, including free amino acids (FAAs), free fatty acids (FFAs), and organic acids, we systematically investigated the temporal changes in these metabolite classes in bovine milk throughout the fermentation process. The targeted metabolomic analysis encompassed the quantification of five organic acids (lactic acid, citric acid, succinic acid, malic acid, and tartaric acid), eight essential FAAs (threonine, valine, methionine, leucine, isoleucine, phenylalanine, lysine, and arginine) and glutamic acid, and six FFAs (oleic acid, capric acid, α-linolenic acid, arachidonic acid, docosahexaenoic acid, and palmitic acid). As illustrated in Figure 5A, the heatmap analysis revealed that organic acids, FAAs, and FFAs were significantly enriched in the CMF5 group compared to the control, indicating enhanced metabolic activity associated with fucose utilisation by *L. helveticus* DYNDL_20-5.

Targeted metabolomic analysis revealed significant differences in the concentrations of multiple organic acids, amino acids, and fatty acids between the CM and CMF5 groups. Specifically, the CMF5 group exhibited significantly elevated levels of succinic acid and malic acid (*p* < 0.01) (Figure 5B,C). Regarding FAAs, statistically significant differences (*p* < 0.05) were detected in the concentrations of eight essential FAAs and glutamic acid between the CM and CMF5 groups (Figure 5D–L). For FFAs, as demonstrated in Figure 5M–O, at the fermentation endpoint, the concentrations of oleic acid, capric acid, and arachidonic acid in the CMF5 group exhibited significant increases (*p* < 0.05) compared to the CM group.

## 3. Discussion

LAB play an essential role in the production of fermented milk. While numerous studies have focused on the effect of LAB for dairy products, relatively few have investigated how modifying the carbon source during fermentation can influence strain-specific metabolism and, consequently, the quality attributes of the final product. Based on the previous finding that *L. helveticus* DYNDL_20-5 produces fucose-containing EPS from fucose, it was hypothesized that culturing this strain with fucose in milk would enhance product quality and metabolism. Thus, the study verifies this hypothesis, demonstrating that fucose supplementation significantly improves both the physicochemical and metabolic characteristics of the fermented milk.

pH is a critical parameter for monitoring fermentation processes and serves as a key indicator for determining fermentation completion. Typically, pH 4.5 is regarded as the threshold value for yogurt fermentation completion [25]. When the pH drops to approximately 4.5, the casein in milk undergoes isoelectric precipitation and coagulates, forming the characteristic gel structure of yogurt whilst simultaneously inhibiting the growth of spoilage and pathogenic bacteria, thereby ensuring food safety [26]. During the first 6 h of fermentation, the rate of pH decline in the CMF5 group was relatively slow, suggesting that *L. helveticus* DYNDL_20-5 may have encountered environmental stresses during the initial adaptation phase [27]. The rapid acid production phase occurred between 6 and 8 h, during which *L. helveticus* DYNDL_20-5 appeared to significantly influence the fermentation process by modulating acid production metabolism through fucose utilisation pathways. This biphasic acidification pattern suggests that the presence of fucose may initially impose metabolic constraints before subsequently enhancing the acid-producing capacity of the strain.

The WHC measured by centrifugation reflects the gel matrix’s capacity to retain water through capillary effects, which is indicative of the protein network’s structural strength [28]. Studies have reported a positive correlation between WHC and EPS production ability [29]. In contrast, a decrease in WHC was observed in the CMF5 group, which may be attributed to alterations in the protein network structure resulting from the metabolic activity of *L. helveticus* DYNDL_20-5 in the presence of fucose [30]. The higher total solids content in this group, attributed to the production of EPS by *L. helveticus* DYNDL_20-5 utilizing fucose, may have resulted in modifications to the gel network structure, leading to easier water loss under centrifugal force [31]. Higher EPS production may lead to an increase in pore size and a more irregular protein network, which could be attributed to electrostatic repulsion between negatively charged EPS and proteins, as well as alterations in intermolecular forces [32]. These structural changes likely reduce the gel’s capacity to retain water, thereby decreasing WHC.

The observed increase in species richness is expected due to the introduction of *L. helveticus* DYNDL_20-5. The Shannon index serves as a quantitative measure reflecting both species richness and evenness within microbial communities [33]. The significant increase in Shannon index indicates that the CMF5 group displays enhanced microbial richness and greater community uniformity in the fermented milk matrix. This suggests that fucose supplementation may influence the relative abundance of bacterial species, potentially modulating community composition. Furthermore, the spatial distribution patterns amongst the fermented milk groups indicate that substantial differences exist in the microbial community structure, with the addition of fucose and *L. helveticus* DYNDL_20-5 driving significant shifts in the overall microbial composition [34]. Additionally, the combined addition of fucose and *L. helveticus* DYNDL_20-5 promoted the proliferation of *Lacticaseibacillus rhamnosus*. *Lacticaseibacillus rhamnosus* represents a well-characterised probiotic strain whose exceptional resistance to acidic and bile salt conditions, coupled with its favourable growth characteristics, enables persistent survival and colonisation within the gastrointestinal tract [35].

Although no statistically significant differences were observed in springiness, resilience, and cohesiveness among the experimental groups, the EPS produced by LAB could influence gel structure through variations in their molecular characteristics [36].

Previous research has established that the viscosity of fermented milk increases significantly with EPS formation and demonstrated a positive correlation with EPS content [37]. At the fermentation end point, the CMF5 group exhibited markedly higher apparent viscosity values compared with both the CMF and CM groups, suggesting that *L. helveticus* DYNDL_20-5 produced substantially greater quantities of EPS when utilising fucose as a substrate, thereby contributing to enhanced viscosity and improved textural properties.

The rheological phenomenon that G′ consistently exceeded G″ across all frequency ranges, primarily originates from the complex network structure formed through multiple bonding mechanisms, including electrostatic repulsions and hydrophobic interactions between casein micelles and various non-covalent associations among all constituents of the fermented milk matrix [38]. Under the specific experimental conditions employed, this protein network enables fermented milk to resist deformation effectively, presenting a solid-like state characterised by predominant elastic properties. This viscoelastic behaviour aligns closely with the established rheological characteristics of fermented dairy products [24,39]. The higher G′ and G″ values observed in the CMF group, indicating that the combined addition of fucose and *L. helveticus* DYNDL_20-5 substantially enhanced the gel strength of fermented milk, thereby improving its structural integrity and textural quality.

In non-targeted metabolomic analysis, a clear metabolic differentiation was observed, indicating that *L. helveticus* DYNDL_20-5 exerts significant influence on the metabolic processes occurring within fermented milk through its capacity to utilise fucose as a substrate, thereby generating distinct metabolic signatures characteristic of each treatment group. Further analysis revealed that the CMF5 group exhibited a substantial increase in differentially abundant metabolites, such as Trp-Met. Studies have shown that Trp-Met exhibits antioxidant activity, primarily acting as a direct radical scavenger, with the presence of Trp and Met residues providing electron/hydrogen donating ability that drives its radical scavenging capacity [40]. As reported by Bertazza et al., yoghurt consumption provides access to bioactive molecules produced during fermentation, including oligopeptides, free amino acids, and biogenic amines [41]. We hypothesise that *L. helveticus* DYNDL_20-5 utilises fucose for fermentation processes, which are accompanied by casein proteolysis and subsequent amino acid liberation [42]. This proteolytic activity likely contributes to the enhanced nutritional value and bioactive peptide content of the fermented milk, potentially conferring additional health benefits beyond those associated with conventional fermentation processes. The metabolic activity of LAB in amino acid transformation predominantly occurs through two enzymatic mechanisms: deamination and decarboxylation reactions. These biochemical processes serve multiple functional roles, enabling LAB to adapt to environmental stresses, enhance the organoleptic properties of fermented foods through flavour compound generation, and support bacterial proliferation and metabolic activity in fermented dairy matrices [43]. The observed enrichment of BCAA biosynthesis pathways suggests that fucose metabolism may enhance the proteolytic system of *L. helveticus* DYNDL_20-5, thereby facilitating more efficient protein degradation and subsequent amino acid liberation during the fermentation process.

Organic acids function as natural antimicrobial preservatives and exert profound influences on multiple quality attributes of fermented dairy products, ranging from sensory characteristics to rheological and structural properties. The organic acid profile in fermented milk originates from multiple sources: endogenous acids present in raw milk, metabolic products generated by LAB during fermentation, and compounds released through lipolytic hydrolysis of milk fat during processing and storage. Lactic acid represents the predominant organic acid in fermented milk systems. However, owing to the complex co-fermentation metabolic activities of LAB consortia, fermented milk also accumulates various secondary organic acids, including formic acid, acetic acid, and propionic acid [44]. Malic acid, functioning as an acidity regulator, exhibits several advantageous properties: it generates minimal metabolic heat, imparts a pleasant taste profile, and facilitates amino acid absorption during metabolic processes [45]. The enhanced malic acid content suggests that fucose supplementation may stimulate the growth and metabolic activity of *L. helveticus* DYNDL_20-5, thereby promoting the enzymatic conversion of malic acid to succinic acid through the reductive branch of the tricarboxylic acid (TCA) cycle during fermentation [46]. Succinic acid serves as a crucial signalling molecule in cellular metabolism, participating in the regulation of oxidative stress responses and modulating inflammatory pathways through its interaction with succinate receptors [47]. The elevated succinic acid levels in fucose-supplemented fermented milk may therefore contribute additional health-promoting properties beyond conventional fermented dairy products.

FAAs represent the hydrolytic products of milk proteins during dairy fermentation, generated through the proteolytic enzyme systems of LAB. Beyond their direct nutritional and health-promoting functions, FAAs serve as precursors for subsequent metabolic transformations in fermented milk, yielding diverse flavour-active compounds, including aldehydes, ketones, amines, alcohols, and sulphur-containing volatiles. This metabolic conversion is particularly pronounced for aromatic amino acids (phenylalanine, tyrosine, and tryptophan) and branched-chain amino acids (valine, leucine, and isoleucine), which undergo transamination and subsequent degradation to generate characteristic flavour compounds [48]. This enhanced FAA liberation can be attributed to two synergistic mechanisms: firstly, protein denaturation induced by thermal processing of milk (pasteurisation), which disrupts tertiary and quaternary protein structures and increases susceptibility to proteolytic cleavage; and secondly, the proteolytic activity of LAB during fermentation, mediated by cell envelope-associated proteinases and intracellular peptidases that sequentially degrade proteins into peptides and subsequently into free amino acids [49]. The liberated amino acids undergo further catabolism, generating metabolic intermediates such as α-ketoacids and acyl-CoA derivatives that enter the TCA cycle, thereby contributing to energy metabolism and biosynthetic processes [50]. Valine, leucine, and isoleucine, collectively designated as BCAAs, possess distinctive metabolic and physiological properties. These essential amino acids have been demonstrated to enhance athletic performance through multiple mechanisms, including stimulation of muscle protein synthesis, reduction of exercise-induced muscle damage, and improvement of exercise capacity. Additionally, BCAAs have shown therapeutic potential in alleviating premature ovarian insufficiency by modulating mitochondrial function and reducing oxidative stress [51]. The significant enrichment of BCAAs in fucose-supplemented fermented milk therefore represents a valuable nutritional enhancement with potential health implications.

FFAs possess diverse biological functions extending beyond their classical role as energy substrates, encompassing the regulation of gene expression in adhesion molecules and macrophages, modulation of cytokine and chemokine production, and activation of G-protein-coupled receptors involved in metabolic regulation. Furthermore, FFAs serve as essential precursors for the generation of volatile flavour compounds in fermented dairy products through enzymatic and chemical transformations, including β-oxidation, decarboxylation, and esterification reactions [52]. This elevation in FFA content can be attributed to the lipolytic activity occurring during fermentation, wherein milk fat globule membranes are destabilised and triglycerides are hydrolysed by bacterial lipases and milk lipase, releasing FFAs [53]. Oleic acid, a monounsaturated omega-9 fatty acid, possesses anti-inflammatory properties and contributes to cardiovascular health. Capric acid, a medium-chain fatty acid, exhibits antimicrobial activity and rapid metabolic absorption. Arachidonic acid, an omega-6 polyunsaturated fatty acid, serves as a precursor for eicosanoid synthesis and plays crucial roles in immune function and inflammatory responses.

Collectively, these results demonstrate that *L. helveticus* DYNDL_20-5 possesses the metabolic capacity to utilise fucose for dairy product fermentation, thereby substantially increasing the concentrations of beneficial metabolites in fermented milk, including organic acids with antimicrobial and signalling functions, essential and branched-chain amino acids with nutritional and physiological benefits, and bioactive fatty acids with diverse health-promoting properties. This metabolic enhancement represents a promising strategy for developing functionally improved fermented dairy products with superior nutritional profiles.

## 4. Materials and Methods

### 4.1. Materials

*L. helveticus* DYNDL_20-5 used in this experiment was obtained from the Culture Collection of Food Microorganisms (CCFM) at the Biotechnology Centre of Jiangnan University (Wuxi, China).

Cow milk was procured from KEDI Group (Shangqiu, China); L-fucose was obtained from Yinti Trading Co., Ltd. (Suzhou, China); commercial starter culture powder YO-MIX 505 LYO 200 DCU, containing *Streptococcus thermophilus* and *Lactobacillus delbrueckii subsp. bulgaricus*, was purchased from Danisco China Co., Ltd. (Kunshan, China). All other reagents were purchased from Sinopharm Chemical Reagent Co., Ltd. (Shanghai, China).

### 4.2. Screening Selection

LAB strains obtained from CCFM were preliminarily screened for EPS production based on their “ropy” character, identified by the formation of long strands when extending colonies with an inoculation loop [54]. Among these, strains with positive results were further quantitatively analyzed for EPS content using the phenol-sulfuric acid method to select high producers [55]. Subsequently, the monosaccharide composition of the EPS was determined using a Dionex ICS-5000+ high performance ion chromatography (HPIC) system (Thermo Fisher Scientific, Sunnyvale, CA, USA). A detailed description of the methodology is available in our previous study [17].

### 4.3. Strain Cultivation

After thawing *L. helveticus* DYNDL_20-5 stored at −80 °C, 4% (*v*/*v*) of the culture was inoculated into MRS liquid medium in a laminar flow hood under aseptic conditions. The culture was incubated at 37 °C overnight. Subsequently, streak plating was performed and single colonies were inoculated into fresh liquid medium. After one generation of liquid culture, strain preservation and identification were performed. Following confirmation of accurate identification through 16S rRNA gene sequencing at GENEWIZ Co., Ltd. (Suzhou, China), reactivation was conducted twice to ensure culture viability. After reactivation, the culture was expanded under the same conditions as described above. A plate colony count was performed prior to inoculating fermented milk, and the bacterial suspension concentration was adjusted to 5.0 × 10^7^ CFU·mL^−1^.

### 4.4. Fermented Milk Preparation

Milk fermentation was performed according to the method described by Sun et al. with minor modifications [56]. Raw milk was heated in a water bath to 60 °C, and 0.5% (*w*/*w*) fucose powder was added with continuous stirring for 15 min until completely dissolved. The mixture was then subjected to high-pressure homogenization (63 °C, 20 MPa). The homogenized dairy product was pasteurized at 85 °C for 30 min, rapidly cooled to 37 °C. Three experimental groups were prepared: the CM group (control), which received no fucose and was inoculated with 0.1% (*w*/*w*) commercial starter culture powder only; the CMF group, which was supplemented with fucose and inoculated with the same dose of commercial starter culture; and the CMF5 group, which was supplemented with fucose and inoculated with both the commercial starter culture and 0.02% (*v*/*v*) *L. helveticus* DYNDL_20-5 cell suspension, prepared from activated cultures. All inoculations were performed in a laminar flow hood under aseptic conditions.

For each group, 200 mL of the inoculated milk was placed into a 250 mL blue-capped bottle and fermented at 42 °C for 8 h. Upon reaching the fermentation endpoint, the bottles were immediately transferred to a water bath and cooled to 20 °C to terminate fermentation. Samples were collected at 2 h intervals throughout the fermentation period, and all experiments were performed in triplicate to ensure reproducibility.

### 4.5. Determination of pH and Titratable Acidity

The pH of fermented milk samples was measured using a calibrated pH meter (pH FE20, Mettler Toledo, Greifensee, Switzerland). For titratable acidity (TA) determination, 5 g of fermented milk was thoroughly mixed with 40 mL of boiled and cooled distilled water. Following the addition of 0.5% phenolphthalein indicator, the mixture was titrated with 0.1 mol/L NaOH solution to the endpoint. The titratable acidity was expressed in degrees Thorner (°T) according to the method described by Sun et al. [57].

### 4.6. Absolute Quantification of Bacterial 16S rRNA Amplicon Sequencing

Absolute quantification of bacterial populations was performed by Majorbio BIOTREE Co., Ltd. (Shanghai, China). Briefly, genomic DNA was extracted from fermented milk microorganisms using standard protocols. For absolute quantification, twelve synthetic spike-in sequences with four concentration gradients (10^3^–10^6^ copies) were added to each sample prior to DNA extraction. These spike-in sequences contain conserved regions identical to the native 16S rRNA genes and artificial variable regions with no significant homology to sequences in public databases, thereby serving as references for quantification. The hypervariable V3-V4 region of the bacterial 16S rRNA gene was amplified using the primer pair 338F (5′-ACTCCTACGGGAGGCAGCAG-3′) and 806R (5′-GGACTACHVGGGTWTCTAAT-3′) [58] in a T100 Thermal Cycler PCR system (Bio-Rad, Hercules, CA, USA). Amplicons were subjected to paired-end sequencing on an Illumina Nextseq2000 platform (Illumina, San Diego, CA, USA), followed by bioinformatic processing and analysis of the resulting sequence data. The spike-in reads were used to generate standard curves, enabling the conversion of OTU read counts to absolute abundances per gram of sample.

### 4.7. Determination of Water Retention Capacity

Water retention capacity (WHC) was determined by centrifugation. A 10 g aliquot of fermented milk sample was centrifuged at 6000 rpm (5000× *g*) for 20 min at 4 °C using a Sorvall ST 8R centrifuge (Thermo Fisher Scientific, Waltham, MA, USA). The supernatant was carefully removed, and the remaining sediment was weighed. Each sample was analysed in triplicate. The WHC was calculated using the following equation: WHC (%) = [Sediment Mass (g)/Initial Sample Mass (g)] × 100.

### 4.8. Determination of Rheological Properties

Rheological properties during the fermentation process were assessed using a Discovery HR-3 rotational rheometer (TA Instruments, New Castle, DE, USA). Frequency sweep tests were conducted at 42 °C under a constant strain of 0.5% to determine the frequency dependence of the storage modulus (*G*′) and loss modulus (G″). The apparent viscosity of fermented milk was measured in steady-state mode at 4 °C across a shear rate range of 0.1–100 s^−1^ to characterise the flow behaviour of the final product.

### 4.9. Determination of Textural Properties

Textural properties of yogurt samples were evaluated using a TA.XTPlus texture analyser (Stable Micro Systems, Godalming, UK) equipped with a 45 mm diameter A/BE cylindrical probe. Measurements were performed in back-extrusion compression mode, with each sample tested in triplicate. The testing parameters were as follows: pre-test speed, 1.0 mm/s; test speed, 1.0 mm/s; post-test speed, 10.0 mm/s; compression distance, 30 mm; trigger force, auto-5 g. Textural attributes including firmness, consistency, cohesiveness and viscosity index were derived from the resulting force-time curves.

### 4.10. Non-Targeted Metabolomics Analysis of Fermented Dairy Products

Non-targeted metabolomics profiling was conducted by BIOTREE Co., Ltd. (Shanghai, China). Fermented milk samples were extracted under ice-bath conditions using a methanol: acetonitrile mixture (1:1, *v*/*v*) with 15 min of ultrasonication. The extracts were concentrated to dryness under vacuum for 8 h, then reconstituted in acetonitrile:water (6:4, *v*/*v*). Metabolite profiling of cow milk fermentation samples was performed using a Vanquish ultra high performance liquid chromatography (UHPLC) system (Thermo Fisher Scientific, Waltham, MA, USA) coupled to an Orbitrap Exploris 120 mass spectrometer (Thermo Fisher Scientific, Waltham, MA, USA), in both positive and negative ionisation modes to maximise metabolite coverage.

### 4.11. Targeted Assay for Organic Acids, Amino Acids and Fatty Acids

Targeted quantification of organic acids, amino acids and fatty acids was performed by BIOTREE Co., Ltd. (Shanghai, China). Samples were analysed using a 600-multiple reaction monitoring method with liquid chromatography-tandem mass spectrometry (LC-MS/MS). Chromatographic separation was achieved using an H-Class ultra-high-performance liquid chromatography system (Waters Corporation, Milford, MA, USA) equipped with a Waters Atlantis Premier BEH Z-HILIC column (1.7 µm particle size, 2.1 mm × 150 mm). The mobile phase consisted of ultrapure water: acetonitrile (8:2, *v*/*v*; Phase A) and acetonitrile: ultrapure water (9:1, *v*/*v*; Phase B) delivered under gradient elution conditions. Mass spectrometric detection was performed using a Sciex QTrap 6500 Plus triple quadrupole mass spectrometer (Sciex, Framingham, MA, USA). Raw data files generated by LC-MS/MS were processed using SCIEX Analyst Workstation software (version 1.7.2), and metabolite quantification was performed using Biotree BioBud software (version 2.1.4) with reference to authentic standards.

### 4.12. Data Analysis

Data are expressed as mean ± standard error of the mean (SEM). Differences amongst multiple groups were analysed using one-way ANOVA followed by Dunnett’s multiple comparison test. For data collected over multiple time points (pH and TA changes during fermentation), two-way ANOVA with Tukey’s multiple comparison test was used. Statistical significance was defined as *p* < 0.05 (* *p* < 0.05, ** *p* < 0.01, *** *p* < 0.001, **** *p* < 0.0001). Statistical analyses and graphical representations were performed using GraphPad Prism software (version 9.5, GraphPad Software Inc., San Diego, CA, USA).

## 5. Conclusions

This study reveals that *L. helveticus* DYNDL_20-5, a strain capable of producing fucose-containing EPS, can effectively utilize fucose as a metabolic substrate during milk fermentation, leading to significant improvements in both product quality and metabolic profiles. The comprehensive analysis revealed that *L. helveticus* DYNDL_20-5 demonstrated the capacity to accelerate acid production through fucose metabolism, thereby promoting fermentation kinetics and facilitating enhanced microbial growth—particularly augmenting the proliferation of beneficial bacterial populations. Concurrently, fucose metabolism strengthened the elastic gel network structure, consequently improving both the textural properties and physical stability of fermented milk products. To further elucidate the metabolic mechanisms underlying the observed physicochemical alterations, comparative metabolomic profiling was conducted between the control and fucose-supplemented groups. The metabolomic analysis revealed that *L. helveticus* DYNDL_20-5 significantly enhanced the production of beneficial metabolites, including FAAs, FFAs, and organic acids, during fucose utilisation, thereby modulating associated metabolic pathways, particularly those involved in BCAA biosynthesis and the TCA cycle. These findings provide valuable scientific insights into the practical application of *L. helveticus* DYNDL_20-5 in modulating carbon sources to steer strain-specific metabolism, thereby improving fermented milk quality. Future investigations should focus on elucidating the specific enzymatic mechanisms involved in fucose metabolism, evaluating the sensory acceptability of fucose-supplemented fermented products, and assessing the in vivo bioavailability and health benefits of the enriched metabolites in human intervention studies.

## Data Availability

The original contributions presented in the study are included in the article, further inquiries can be directed to the corresponding author.

## Abbreviations

The following abbreviations are used in this manuscript:

LAB	Lactic acid bacteria
EPS	Extracellular polysaccharides
CCFM	Collection of Food Microorganisms
HPIC	high performance ion chromatography
TA	Titratable acidity
WHC	Water holding capacity
UHPLC	Ultra high performance liquid chromatography
LC-MS/MS	Liquid chromatography-tandem mass spectrometry
SEM	Standard error of the mean
ANOVA	Analysis of Variance
OTUs	Operational taxonomic units
PCoA	Principal coordinate analysis
LEfSe	Linear discriminant analysis Effect Size
PCA	Principal component analysis
BCAAs	Branched-chain amino acids
FAAs	Free amino acids
FFAs	Free fatty acids
TCA	Tricarboxylic acid
Trp-Met	Tryptophan-methionine
